# Supplementary material for: Sn1,3 Regiospecificity of DHA (22:6ω-3) of Plant Origin (DHA-Canola®) Facilitates Its Preferential Tissue Incorporation in Rats Compared to sn2 DHA in Algal Oil at Low Dietary Inclusion Levels
Source: Nutrients. 2025 Apr 9;17(8):1306. doi: 10.3390/nu17081306 (PMC12030648; doi:10.3390/nu17081306)
Supplement: Supplementary file 1 [file nutrients-17-01306-s001.zip › nutrients-3205269 - Supplementary Table S1.pdf]

Supplementary Table S1. Composition of experimental diets (g per Kg) [1].

Ingredient	HOSO	DHA-Control				DHA-Canola			
		0.3 %	1.0 %	3.0 %	6.0 %	0.3 %	1.0 %	3.0 %	6.0 %
**Cornflour**	529.5	529.5	529.5	529.5	529.5	529.5	529.5	529.5	529.5
**Casein**	200	200	200	200	200	200	200	200	200
**Sucrose**	100	90	90	90	90	90	90	90	90
**HOSO**	70	67	60	40	10	67	60	40	10
**DHA-Canola**	0	0	0	0	0	3	10	30	60
**DHA-Control**	0	3	10	30	60	0	0	0	0
**α- cellulose**	50	50	50	50	50	50	50	50	50
**Vitamin mix AIN-93G**	10	10	10	10	10	10	10	10	10
**Mineral mix AIN-93G**	35	35	35	35	35	35	35	35	35
**L-Cystine**	3	3	3	3	3	3	3	3	3
**Choline Chloride**	2.5	2.5	2.5	2.5	2.5	2.5	2.5	2.5	2.5

[1] The DHA-Control oil was a blend of Docosahexaenoic acid single cell oil (DHASCO) and high oleic sunflower seed oil (HOSO) to yield a comparable DHA level to that of DHA-Canola.
