# Supplementary material for: Sn1,3 Regiospecificity of DHA (22:6ω-3) of Plant Origin (DHA-Canola®) Facilitates Its Preferential Tissue Incorporation in Rats Compared to sn2 DHA in Algal Oil at Low Dietary Inclusion Levels
Source: Nutrients. 2025 Apr 9;17(8):1306. doi: 10.3390/nu17081306 (PMC12030648; doi:10.3390/nu17081306)
Supplement: Supplementary file 1 [file nutrients-17-01306-s001.zip › nutrients-3205269 - Supplementary Table S2.pdf]

Supplementary Table S2. Organ weights (g)[1,2].

	Heart	Liver	Kidney	Brain	Spleen	Thymus
**HOSO**	1.5 ± 0.1	16.0 ± 0.8	2.6 ± 0.1	1.8 ± 0.0	0.9 ± 0.0	0.5 ± 0.1
**DHA-Control**						
**0.3%**	1.6 ± 0.1	15.5 ± 0.4	2.7 ± 0.1	1.9 ± 0.0	1.0 ± 0.1	0.4 ± 0.0
**1.0%**	1.6 ± 0.1	15.7 ± 0.4	2.7 ± 0.1	1.9 ± 0.0	1.0 ± 0.0	0.4 ± 0.0
**3.0%**	1.5 ± 0.1	15.1 ± 0.5	2.7 ± 0.1	1.8 ± 0.0	0.9 ± 0.0	0.4 ± 0.0
**6.0%**	1.6 ± 0.1	16.5 ± 0.6	2.8 ± 0.1	1.8 ± 0.0	1.0 ± 0.0	0.5 ± 0.1
**DHA-Canola**						
**0.3%**	1.5 ± 0.0	15.0 ± 0.4	2.7 ± 0.1	1.8 ± 0.0	1.0 ± 0.0	0.4 ± 0.0
**1.0%**	1.5 ± 0.1	16.2 ± 0.7	2.8 ± 0.1	1.8 ± 0.0	1.0 ± 0.0	0.4 ± 0.0
**3.0%**	1.5 ± 0.0	15.8 ± 0.5	2.8 ± 0.1	1.8 ± 0.0	1.0 ± 0.0	0.4 ± 0.0
**6.0%**	1.5 ± 0.1	15.5 ± 0.8	2.8 ± 0.1	1.8 ± 0.0	1.0 ± 0.1	0.4 ± 0.0

[1] Values are expressed as mean ± SEM for n = 8 animals per group. [2] Dietary treatments did not affect organ weights (ANOVA, Tukey's at $p < 0.05$). HOSO, high oleic sunflower seed oil.
