# Supplementary material for: Sn1,3 Regiospecificity of DHA (22:6ω-3) of Plant Origin (DHA-Canola®) Facilitates Its Preferential Tissue Incorporation in Rats Compared to sn2 DHA in Algal Oil at Low Dietary Inclusion Levels
Source: Nutrients. 2025 Apr 9;17(8):1306. doi: 10.3390/nu17081306 (PMC12030648; doi:10.3390/nu17081306)
Supplement: Supplementary file 1 [file nutrients-17-01306-s001.zip › nutrients-3205269 - Supplementary Table S3.pdf]

Supplementary Table S3. Brain phospholipid fatty acid composition (% total fatty acids)[1].

Fatty Acid	HOSO	0.3% DHA		1.0% DHA		3.0% DHA		6.0% DHA	
		Control	Canola	Control	Canola	Control	Canola	Control	Canola
20:5ω3	n.d.	n.d.	n.d.	0.01	0.04*	0.1	0.2	0.3	0.3
22:5ω3	0.01	0.01	0.04*	0.1	0.1	0.1	0.3	0.3	0.4
22:6ω3	16.2	19.5	19.1	18.8	18.7	20.8	19.2	21.8	19.4
Σ ω3 PUFA	16.3	19.5	19.1	18.9	18.8	21.0	19.6	22.5	20.1
Σ ω6 PUFA	17.0	16.5	16.0	13.9	13.6	13.3	12.3	13.0	12.4
Σ ω-3/ω-6	1.0	1.2	1.2	2.7	1.4	1.5	1.6	1.7	1.7

[1]At each supplementation level, significant differences between Control (DHA-Control) and Canola (DHA-Canola) are shown as * $p < 0.05$ (student unpaired *t*-test). Mean values (n = 7–8). HOSO, high oleic sunflower seed oil.
